# Long-term exposure to particulate matter is associated with elevated blood pressure: Evidence from the Chinese plateau area

**DOI:** 10.7189/jogh.14.04039

**Published:** 2024-03-15

**Authors:** Yajie Li, Bin Yu, Li Yin, Xianzhi Li, Qucuo Nima

**Affiliations:** 1 Tibet Centre for Disease Control and Prevention, Lhasa, Tibet Autonomous Region, China; 2 Institute for Disaster Management and Reconstruction, Sichuan University – Hong Kong Polytechnic University, Chengdu, China; 3 West China School of Public Health and West China Fourth Hospital, Sichuan University, Chengdu, China; 4 Meteorological Medical Research Center, Panzhihua Central Hospital, Panzhihua, China; 5 Clinical Medical Research Center, Panzhihua Central Hospital, Panzhihua, China; 6 Dali University, Dali, China

## Abstract

**Background:**

Ambient air pollution could increase the risk of hypertension; however, evidence regarding the relationship between long-term exposure to particulate matter and elevated blood pressure in plateau areas with lower pollution levels is limited.

**Methods:**

We assessed the associations of long-term exposure to particulate matter (PM, PM_1_, PM_2.5_, and PM_10_) with hypertension, diastolic blood pressure (DBP), systolic blood pressure (SBP) and pulse pressure (PP) in 4.235 Tibet adults, based on the baseline of the China multi-ethnic cohort study (CMEC) in Lhasa city, Tibet from 2018–19. We used logistic regression and linear regression models to evaluate the associations of ambient PM with hypertension and blood pressure, respectively.

**Results:**

Long-term exposure to PM_1_, PM_2.5_, and PM_10_ is positively associated with hypertension, DBP, and SBP, while negatively associated with PP. Among these air pollutants, PM_10_ had the strongest effect on hypertension, DBP, and SBP, while PM_2.5_ had the strongest effect on PP. The results showed for hypertension odds ratio (OR) = 1.99; 95% confidence interval (CI) = 1.58, 2.51 per interquartile range (IQR) μg/m^3^ increase in PM_1_, OR = 1.93; 95% CI = 1.55, 2.40 per IQR μg/m^3^ increase in PM_2.5_, and OR = 2.12; 95% CI = 1.67, 2.68 per IQR μg/m^3^ increase in PM_10_.

**Conclusions:**

Long-term exposure to ambient air pollution was associated with an increased risk of hypertension, elevated SBP and DBP levels, and decreased PP levels. To reduce the risk of hypertension and PP reduction, attention should be paid to air quality interventions in plateau areas with low pollution levels.

Hypertension, or high blood pressure (BP), is a major cause of premature death worldwide in the 21st century. Despite being asymptomatic, it is a significant risk factor for cardiovascular disease, including stroke, coronary artery disease, heart failure, and atrial fibrillation [1–4]. In 2010, it was estimated that 1.39 billion people worldwide had hypertension, with a higher prevalence in low and middle-income countries than in high-income countries [5]. In China, the weighted prevalence of hypertension in individuals aged 18 years or above was 23.2% [6]. As research progressed, epidemiological studies have demonstrated different profiles of hypertension across socio-demographic characteristics, such as place of residence, age, and sex [7–9]. For example, previous studies in Chinese Tibet, one of the highest inhabited areas of the world, suggested a higher hypertension rate than in other Chinese areas [10,11], as well as non-Chinese regions where inhabitants live at high altitudes [12]. Although causative studies have indicated the significant role of high altitude in hypertension development [13], there is a need for in-depth research on hypertension prevention and control among residents of high-altitude areas [9,11].

Numerous observational studies have investigated modifiable environmental determinants of hypertension, among which air pollutants have been identified as a crucial risk factor [14–16]. For example, one study conducted in seven northeast cities in China found that exposure to airborne particulates with an aerodynamic diameter ≤2.5μm (PM_2.5_) was significantly and positively associated with systolic blood pressure (SBP) and hypertension [16]. Another study based on the UK Biobank data suggested that five major air pollutants were positively associated with subsequent hypertension [9]. A prospective cohort study recruiting 74 880 participants attending the prospective nationwide Nurses’ health study suggested that PM_2.5_ and PM≤10 μm (PM_10_) were associated with small increases in the incidence of hypertension [17]. These data provide solid evidence that ambient air pollutants are important triggering factors for hypertension development and cardiovascular events.

Most previous studies have focused solely on participants in plain regions to estimate the association between ambient air pollutants and hypertension [18,19], which may have resulted in a limited understanding of the comprehensive impacts of ambient air pollution on hypertension among residents of high-altitude areas [20,21]. Intuitively, individuals residing at higher altitudes appear to face a lower risk of hypertension, as they are exposed to lower levels of air pollution compared to those living at lower altitudes. However, medical problems (e.g. hypoxic pulmonary vasoconstriction) and cardiovascular events occur at high altitudes due to low inspired oxygen and the relatively unique lifestyle of indigenous people (e.g. dietary pattern with excessive intake of sodium) [8,22]. Considering that plateau environments are significantly different from plains environments, gaining an insight into the air pollutants-hypertension mechanism may aid in preventing and controlling hypertension through the management of air pollution emissions in plateau areas.

We aimed to investigate the association of ambient air pollution with hypertension among residents of Tibet, known as the world’s ‘Third Pole’. The findings provide evidence for multiple stakeholders, including environmental policymakers and health institutions, to develop effective measures for preventing hypertension among residents of high-altitude areas.

## METHODS

### Study population

The study was part of the China multi-ethnic cohort study (CMEC) conducted from May 2018 to September 2019 [23]. Data used in the study was from Tibet, one of five provinces in CMEC, located southwest of the Qinghai-Tibet Plateau, China. We selected participants by multi-stage stratified cluster sampling. First, we selected the Chengguan district of Lhasa in Tibet as the research site. Second, we selected five communities from the 12 communities in Chengguan district by local health institutions, considering their health condition and immigration status. Third, we invited all residents in the study sites to participate, which resulted in 7737 potential participants. For the current analyses, we excluded participants with incomplete addresses, who lived at the current address for less than three years on the day of investigation, who had any physician-diagnosed hypertension (considering that hypertensive patients may take medication to control their blood pressure), participants with missing information on any outcome, exposure, or adjusted covariates, and participants age less than 18 years old. Ultimately, a total of 4235 eligible participants remained in the analyses (Figure S1 in the **Online Supplementary Document**).

### Air pollution exposure assessment

Daily PM_1_, PM_2.5_, and PM_10_ were estimated by Wei et al. at ‘1 km ×1 km’ spatial resolution using a space-time extremely randomised trees model based on monitoring data, meteorology, land use information, pollution emissions and other spatial and temporal predictors [24–26]. We obtained air pollution data during the study period from the China High Air Pollutants data set, and the relevant research results have shown a high predictive ability [24–26]. We calculated the three-year average concentrations of PM_1_, PM_2.5_, and PM_10_ for each participant based on their residential address.

### Outcome and covariates assessment

The outcomes in the current study included hypertension, SBP, diastolic blood pressure (DBP), and pulse pressure (PP). We defined hypertension as having an average measured SBP≥140 mm Hg and/or DPB≥90 mm Hg according to the 2017 American College of Cardiology/American Heart Association hypertension guideline [27]. According to the American Heart Association’s standardised protocol, trained medical personnel measured participants’ BP using electronic sphygmomanometers [28]. Participants were told not to smoke, drink alcohol, drink coffee or tea, or exercise for at least 30 minutes before the measurement. All BP measurements were performed in a seated, upright position three times. We calculated SBP and DBP based on the average of the three measurements. Further, we calculated PP as the difference between SBP and DBP.

The covariates included demographic characteristics (sex, age, and marital status), socioeconomic gradient (annual family income and education level), health behaviours (smoking status, secondary smoking, alcohol drinking status, physical activity, and dietary approaches to stop hypertension (DASH) score), health-related variable (hypertension family history and body mass index (BMI)), and indoor air pollution. We defined current smoking as a total of more than 100 cigarettes smoked to date, and former smoking was defined as quitting for more than half a year [29]. We defined secondary smoking as whether a person had a history of passive smoking at home or in public places. Further, we defined often drinking as drinking alcohol more than two days per week on average over the past year, while occasional drinking was defined as drinking alcohol less than three days per week on average during the past year. The DASH score focused on seven kinds of foods, including vegetables, fresh fruits, legumes, whole grains, red and processed meat, dairy, and sodium salt, and each kind of food was assigned a score of one to five according to the quintile of the average food intake [30,31], with a total score ranging from seven to 35. Physical activity was estimated by the international physical activity questionnaire [32], considering participants’ occupational, transport, household, and leisure physical activities [33]. We assigned metabolic equivalent values (MET) to estimate the physical activity intensity [33]. We calculated the product of physical activity intensity (i.e. MET), and duration (hour/d) as the volume of activity (MET – hour/d). Indoor air pollution considered cooking frequency, fuel usage, and ventilation equipment and was classified as low, moderate, and high based on relevant literature [34].

### Statistical methods

We used descriptive statistics for sample characteristics under the normotension group and hypertension group. Continuous and categorical characteristic variables were presented as means, standard deviation (SD), and numbers (percentages), and were compared using Student’s *t* test and χ^2^ test, respectively.

We measured the participants’ blood pressure using continuous (e.g. SBP, DBP, and PP) and dichotomous variables (e.g. hypertension). Based on previous research [34], multivariable logistic regression models were used to evaluate the association between long-term exposure to PM_1_, PM_2.5_, and PM_10_ and the risk of hypertension. We used linear regression models to assess the association between PM and SBP, DBP, and PP. Based on the previous literature on air pollution and hypertension [34], fully adjusted models included covariates of age, sex, marital, annual family income, educational level, smoking, secondary smoking, alcohol drinking, physical activity, hypertension family history, BMI, DASH score, and indoor air pollution. The estimated effects were represented by odds ratio (ORs) with 95% confidence intervals (CIs) or β with 95% CIs. In addition, we used restricted cubic spline analysis to test the potential nonlinear relationships between PM and hypertension, SBP, DBP, and PP [35].

We performed a series of sensitivity analyses. First, we used one-year, two-year, and four-year average ambient PM concentrations to fit the adjusted models. Second, we adjusted for three-year average temperature and humidity to minimise the influence of environmental factors. Third, we adjusted for diabetes to minimise the influence of comorbidity. All statistical analyses were performed using *R*, version 4.2.2 (R Core Team, Vienna, Austria), and statistical significance was declared as *P*-value <0.05.

## RESULTS

Among the 4235 participants included in this study, the mean age was 45.21 years (SD = 12.44), and 61.18% were female. Normotensive participants were more likely to be female and younger, with higher annual family income, higher education level, non-smokers, non-drinkers, and had a higher level of physical activity and higher DASH score. The three-year average concentrations of PM_1_, PM_2.5_, and PM_10_ were 14.14, 22.06, and 61.54, respectively. In general, participants with hypertension tended to have higher PM_1_, PM_2.5_, and PM_10_ exposures than normotensive participants (**Table 1** and **Table 2**).

**Table 1:** Basic characteristics of study participants*

Characteristics	Total	Normotension	Hypertension	*P*-value
Age in years, x̄ (SD)	45.21 (12.44)	44.09 (12.15)	53.08 (11.59)	<0.001
Sex				<0.001
*Male*	1644 (38.82)	1387 (37.41)	257 (48.77)	
*Female*	2591 (61.18)	2321 (62.59)	270 (51.23)	
Marital status				0.200
*Married/cohabitating*	3640 (85.95)	3177 (85.68)	463 (87.86)	
*Unmarried/divorced/widowed*	595 (14.05)	531 (14.32)	64 (12.14)	
Annual family income, yuan/y (%)				0.002
*<12 000*	872 (20.59)	738 (19.90)	134 (25.43)	
*12 000–19 999*	1182 (27.91)	1020 (27.51)	162 (30.74)	
*20 000–59 999*	1481 (34.97)	1323 (35.68)	158 (29.98)	
*≥60 000*	700 (16.53)	627 (16.91)	73 (13.85)	
Education level				<0.001
*Illiteracy*	2103 (49.66)	1809 (48.79)	294 (55.79)	
*Primary school*	1272 (30.04)	1110 (29.94)	162 (30.74)	
*Junior high school and above*	860 (20.31)	789 (21.28)	71 (13.47)	
Smoking status				<0.001
*Never*	3205 (75.68)	2831 (76.35)	374 (70.97)	
*Former*	189 (4.46)	145 (3.91)	44 (8.35)	
*Current*	841 (19.86)	732 (19.74)	109 (20.68)	
Secondary smoking				0.020
*No*	3185 (75.21)	2767 (74.62)	418 (79.32)	
*Yes*	1050 (24.79)	941 (25.38)	109 (20.68)	
Alcohol drinking status				<0.001
*Never*	2812 (66.40)	2499 (67.39)	313 (59.39)	
*Occasionally*	1158 (27.34)	1016 (27.40)	142 (26.94)	
*Often*	265 (6.26)	193 (5.20)	72 (13.66)	
Physical activity, MET – hour/d (SD)	21.51 (16.96)	21.78 (17.23)	19.62 (14.80)	0.050
DASH score, x̄ (SD)	20.67 (3.39)	20.71 (3.39)	20.37 (3.40)	0.035
Hypertension family history				<0.001
*No*	1739 (41.06)	1523 (41.07)	216 (40.99)	
*Not sure*	1512 (35.70)	1289 (34.76)	223 (42.31)	
*Yes*	984 (23.23)	896 (24.16)	88 (16.70)	
BMI, kg/m^2^ (%)				<0.001
*0–23.99*	1407 (33.22)	1305 (35.19)	102 (19.35)	
*24.00–27.99*	2194 (51.81)	1892 (51.02)	302 (57.31)	
*≥28.00*	634 (14.97)	511 (13.78)	123 (23.34)	
Indoor air pollution				0.360
*Low*	557 (13.15)	479 (12.92)	78 (14.80)	
*Moderate*	3363 (79.41)	2948 (79.50)	415 (78.75)	
*High*	315 (7.44)	281 (7.58)	34 (6.45)	
Three-year average PM_1_, μg/m^3^ (SD)	14.73 (2.50)	14.66 (2.47)	15.25 (2.61)	<0.001
Three-year average PM_2.5_, μg/m^3^ (SD)	24.57 (6.11)	24.39 (6.05)	25.83 (6.36)	<0.001
Three-year average PM_10_, μg/m^3^ (SD)	66.38 (16.27)	65.86 (16.04)	70.09 (17.39)	<0.001

x̄ – mean, BMI – body mass index, DASH – dietary approaches to stop hypertension, METs – metabolic equivalent tasks, PM_1_ – particle with an aerodynamic diameter of 1 μm or less, PM_2.5_ – particle with an aerodynamic diameter of 2.5 μm or less, PM_10_ – particle with an aerodynamic diameter of 10 μm or less, SD – standard deviation

*Presented as n (%) unless specified otherwise.

**Table 2:** Three-year average concentrations of ambient air pollutants

Variables	Minimum	Maximum	P_25_	P_50_	P_75_	IQR
PM_1_	10.38	30.78	11.99	14.14	18.05	6.06
PM_2.5_	17.07	46.65	18.42	22.06	32.44	14.03
PM_10_	38.73	103.92	49.36	61.54	89.11	39.74

IQR – interquartile range, P_25_ – 25th percentile, P_50_ – 50th percentile, P_75_ – 75th percentile PM_1_ – particle with an aerodynamic diameter of 1 μm or less, PM_2.5_ – particle with an aerodynamic diameter of 2.5 μm or less, PM_10_ – particle with an aerodynamic diameter of 10 μm or less

Long-term exposure to PM was positively associated with hypertension, SBP, and DBP, while negatively associated with PP. The correlation between them was relatively stable in all three models, and results were similar after adjusting for other factors (**Table 3** and Table S1 in the **Online Supplementary Document**).

**Table 3:** Associations of risk of hypertension with per 10 μg/m^3^ increase in ambient air pollutants

Outcome	Model 1*	Model 2†	Model 3‡
Hypertension, OR (95% CI)			
*PM_1_*	2.868 (1.973, 4.169)	3.100 (2.116, 4.542)	3.118 (2.127, 4.571)
*PM_2.5_*	1.553 (1.333, 1.809)	1.592 (1.361, 1.862)	1.595 (1.363, 1.866)
*PM_10_*	1.194 (1.126, 1.265)	1.206 (1.137, 1.280)	1.208 (1.138, 1.282)
SBP, β (95% CI)			
*PM_1_*	2.962 (1.328, 4.596)	3.000 (1.379, 4.621)	2.991 (1.369, 4.613)
*PM_2.5_*	1.176 (0.508, 1.844)	1.130 (0.465, 1.795)	1.128 (0.462, 1.793)
*PM_10_*	0.550 (0.300, 0.801)	0.548 (0.299, 0.797)	0.547 (0.298, 0.796)
DBP, β (95% CI)			
*PM_1_*	4.409 (3.221, 5.596)	4.471 (3.296, 5.645)	4.477 (3.302, 5.651)
*PM_2.5_*	1.953 (1.468, 2438)	1.940 (1.458, 2.421)	1.944 (1.462, 2.425)
*PM_10_*	0.781 (0.599, 0.963)	0.784 (0.604, 0.964)	0.784 (0.604, 0.964)
PP, β (95% CI)			
*PM_1_*	−1.432 (−2.527, −0.337)	−1.456 (−2.561, −0.352)	−1.472 (−2.576, −0.368)
*PM_2.5_*	−0.772 (−1.219, −0.324)	−0.804 (−1.257, −0.352)	−0.810 (−1.263, −0.358)
*PM_10_*	−0.228 (−0.396, −0.060)	−0.233 (−0.403, −0.064)	−0.235 (−0.405, −0.066)

CI – confidence interval, DBP – diastolic blood pressure, OR – odds ratio, PM_1_ – particle with an aerodynamic diameter of 1 μm or less, PM_2.5_ – particle with an aerodynamic diameter of 2.5 μm or less, PM_10_ – particle with an aerodynamic diameter of 10 μm or less, PP – pulse pressure, SBP – systolic blood pressure

*Model 1 was adjusted for age, sex, marital, annual family income, and educational level.

†Model 2 was further adjusted smoking, secondary smoking, alcohol drinking, physical activity, hypertension family history, and body mass index.

‡Model 3 was further adjusted DASH score and indoor air pollution.

In general, PM_10_ was associated with the largest absolute changes in hypertension, SBP, and DBP, while PM_2.5_ was associated with the largest absolute changes in PP. The associations between hypertension, SBP, DBP, and PP and per IQR μg/m^3^ increase in three-year average ambient air pollutants exposure are shown in **Table 4**.

**Table 4:** Associations of risk of hypertension with per IQR μg/m^3^ increase in ambient air pollutants*

Pollutant	PM_1_	PM_2.5_	PM_10_
Hypertension, OR (95% CI)	1.992 (1.580, 2.512)	1.925 (1.545, 2.399)	2.119 (1.673, 2.683)
SBP, β (95% CI)	1.813 (0.830, 2.795)	1.583 (0.648, 2.516)	2.174 (1.184, 3.163)
DBP, β (95% CI)	2.713 (2.001, 3.425)	2.727 (2.051, 3.402)	3.116 (2.400, 3.831)
PP, β (95% CI)	−0.892 (−1.561, −0.223)	−1.136 (−1.772, −0.502)	−0.934 (−1.609, −0.262)

CI – confidence interval, DBP – diastolic blood pressure, OR – odds ratio, PM_1_ – particle with an aerodynamic diameter of 1 μm or less, PM_2.5_ – particle with an aerodynamic diameter of 2.5 μm or less, PM_10_ – particle with an aerodynamic diameter of 10 μm or less, PP – pulse pressure, SBP – systolic blood pressure

*Model was adjusted for age, sex, marital, annual family income, educational level, smoking, secondary smoking, alcohol drinking, physical activity, hypertension family history, body mass index, DASH score, and indoor air pollution.

The sensitivity analysis showed comparable effect estimates for hypertension, SBP, DBP, and PP when using the average ambient air pollution concentrations from years before the survey as the exposure variable. According to the results of the restricted cubic spline models, the relationships between long-term PM exposure and hypertension, SBP, DBP, and PP were nonlinear (*P* < 0.05) (Table S2 and Figure S2 in the **Online Supplementary Document**).

## DISCUSSION

Our study found that long-term exposure to ambient PM_1_, PM_2.5_, and PM_10_ was associated with an increased prevalence of hypertension, elevated SBP and DBP levels, and decreased PP levels among Tibetan people in plateau and low-pollution areas. There were nonlinear associations between PM and hypertension, DBP, SBP, and PP. The association remained robust after adjusting for potential confounders, such as demographic characteristics, socioeconomic status, and health status. We also found that long-term exposure to PM_10_ had the greatest effect on the risk of hypertension among the three ambient PMs. To our knowledge, this is the first study to examine the associations between PM and hypertension as well as BP among Tibet adults in the plateau area.

Our findings indicated that long-term exposures to PM_1_, PM_2.5_, and PM_10_ were all positively related to increased risk of hypertension and elevated levels of BP. The results were consistent with most previous studies [19,34,36–39]. A study from China that included 99 084 adults from three cohorts found that long-term exposure to PM2.5 was associated with increased SBP and DBP levels and the risk of hypertension [40]. Results from the CMEC revealed that high PM (including PM_1_, PM_2.5_, and PM_10_) exposure is associated with an increased prevalence of hypertension and increased BP [34]. A meta-analysis in 2021 reported that the risk of hypertension was significantly increased in adults with each 10 μg/m^3^ increase in exposure to PM_2.5_ (OR = 1.10; 95% CI = 1.07, 1.14), PM_10_ (OR = 1.04; 95% CI = 1.02, 1.07) [41]. Another recently published meta-analysis included 41 studies and showed a positive association between long-term exposure to PM and increased BP and hypertension [42]. However, most of the current studies from China were conducted among the non-Tibetan population in non-plateau areas with relatively high PM concentration, which made it difficult to extrapolate their results to the Tibetan population in the plateau area due to the disparities of genetic background, demographic characteristics, health-related behaviour and PM components. The effects of long-term PM on BP and hypertension in studies conducted in China tended to be higher than our results. For example, a study conducted in Southwest China found that the effect estimates of hypertension were OR = 1.109 (95% CI = 1.027, 1.198), OR = 1.101 (95% CI = 1.055, 1.148), and OR = 1.053 (95% CI = 1.022, 1.084) per 10 μg/m^3^ increase in PM_1_, PM_2.5_, and PM_10_, respectively [34]. By comparison, our study observed a higher effect size between PM and hypertension, in which OR = 3.118; 95% CI = 2.127, 4.571, OR = 1.595; 95%CI = 1.363, 1.866), 1.208 (95% CI = 1.138, 1.282) per 10 μg/m^3^ increase in PM_1_, PM_2.5_, and PM_10_, respectively. Nevertheless, the positive association between PM and BP and hypertension remains inconclusive, and some studies found a statistically non-significant association [43–47]. In a nationwide study of Chinese adults aged 35 and older, Liu et al. found significant associations between PM_2.5_ and SBP but not DBP [47]. A study that included 4121 older Americans found a correlation between long-term exposure to PM_2.5_ and SBP, but not DBP [18]. Yet another study conducted in Taipei observed isolated elevations in DBP, but not SBP, with one year of exposure to air pollution among elderly participants [44]. Several factors may contribute to this inconsistency, including differences in PM concentration and composition across study regions and population variations. Besides, our study found that long-term exposure to ambient PM was associated with decreased levels of PP, which is inconsistent with other research findings [48]. This may be due to the fact that the elevating effect of PM on DBP is stronger than that on SBP. The negative association with PP is less common and warrants further investigation.

Several potential biological pathways have been proposed in observational and experimental studies as explanations for elevated BP caused by PM [49,50]. First, PM may cause an imbalance between sympathetic and parasympathetic tone in the autonomic nervous system, and this effect may be triggered by lung irritant sensory receptors and afferents [51,52]. Additionally, PM breathing could stimulate the production and release of vascular-active molecules and proinflammatory mediators from various sources (especially lung cells) that have been shown to ‘spill over’ into the systemic circulation [51,53]. Particles inhaled into the lungs are supposed to initiate an inflammatory response in the alveolae, which in turn results in systemic inflammation that damages the arteries [50]. Third, soluble constituents (e.g. metal) and/or nano-sized particles may be able to penetrate the alveolar membrane into the bloodstream and directly affect the vascular endothelium [54–57]. In summary, PM can mediate an increase in BP in a biphasic manner, including an initial response within minutes to hours (due to acute autonomic nervous system imbalance) and a subsequent increase in BP due to increased arterial vasoconstrictor reactivity (because of endothelial dysfunction, oxidative stress, and inflammation) [49]. With increased elevation, the temperature and partial pressure of oxygen are reduced, resulting in a hypobaric hypoxic environment. The high-altitude cold and hypoxic environment affects the body’s respiratory function, mainly manifested as tachypnoea and increased lung ventilation volume [58,59]. This change in respiratory function may increase the inhalation of air pollutants due to higher ventilation, which could amplify the adverse health effects of air pollutants [60].

Different sizes of particles have different toxic substances attached to their surfaces and can access different parts of the body, which causes different health effects [61]. The reported ORs and CIs indicate a significant association, but the effect sizes vary across different PM types, suggesting differential impacts. Among the three PM fractions, we found that PM_10_ had the largest effect estimates compared to PM_1_ and PM_2.5_. The results were inconsistent with the assumption in previous studies that smaller particles can penetrate the deep respiratory tract, have a higher surface volume ratio, and carry more toxins, which lead to more severe oxidative stress and inflammation [62,63]. The reason for this inconsistency may be related to the composition of different particle sizes and the genetic background of the population.

Our study has several strengths. First, an extensive set of covariates (i.e. indoor air pollution) were incorporated into the analysis so as to control for confounding factors. Second, the concentrations of air pollutants in this study were relatively lower, and this study was conducted in a plateau area, so it could further provide an understanding of PM-related health effects in low-pollution areas of the plateau. Nevertheless, there were also several limitations. First, due to the inherent limitations of cross-sectional studies, making causal inferences between PM, hypertension, and BP should be cautious. Second, the definition of hypertension based on a single visit measurement might not be as accurate as multiple measurements over time. Third, although the main models have adjusted for indoor air pollution factors, the individual exposure variation to indoor and outdoor air pollution has not been considered, which may affect the accuracy of the results. Fourth, our participants were all Tibetans from a plateau area in China who have unique geographic and demographic characteristics, so the findings of this study might not be generalisable to other populations. Fifth, due to the limited availability of data, it was impossible to adjust for all possible confounding variables. Finally, part of the information in this study, such as physical activity, is self-reported, which may result in recall bias.

## CONCLUSIONS

Long-term exposure to ambient air pollution was associated with an increased risk of hypertension, elevated levels of SBP and DBP, and decreased levels of PP. Among these air pollutants, PM_10_ had the strongest effect on hypertension, DBP, and SBP, while PM_2.5_ had the strongest effect on PP. Environmental policymakers and multiple stakeholders, including health institutions, should pay attention to and develop effective air quality interventions in plateau areas with low pollution levels to reduce the risk of hypertension and PP reduction. Future research could benefit from a longitudinal design and a deeper exploration of the unique environmental and genetic factors at play in these regions.

## Additional material


Online Supplementary Document.
