# Supplementary material for: Long-term exposure to particulate matter is associated with elevated blood pressure: Evidence from the Chinese plateau area
Source: J Glob Health. 2024 Mar 15;14:04039. doi: 10.7189/jogh.14.04039 (PMC10939114; doi:10.7189/jogh.14.04039)
Supplement: Online Supplementary Document. [file jogh-14-04039-s001.pdf]

Supplementary Materials:

Long-term exposure to particulate matter is associated with elevated blood pressure: Evidence from the Chinese plateau area

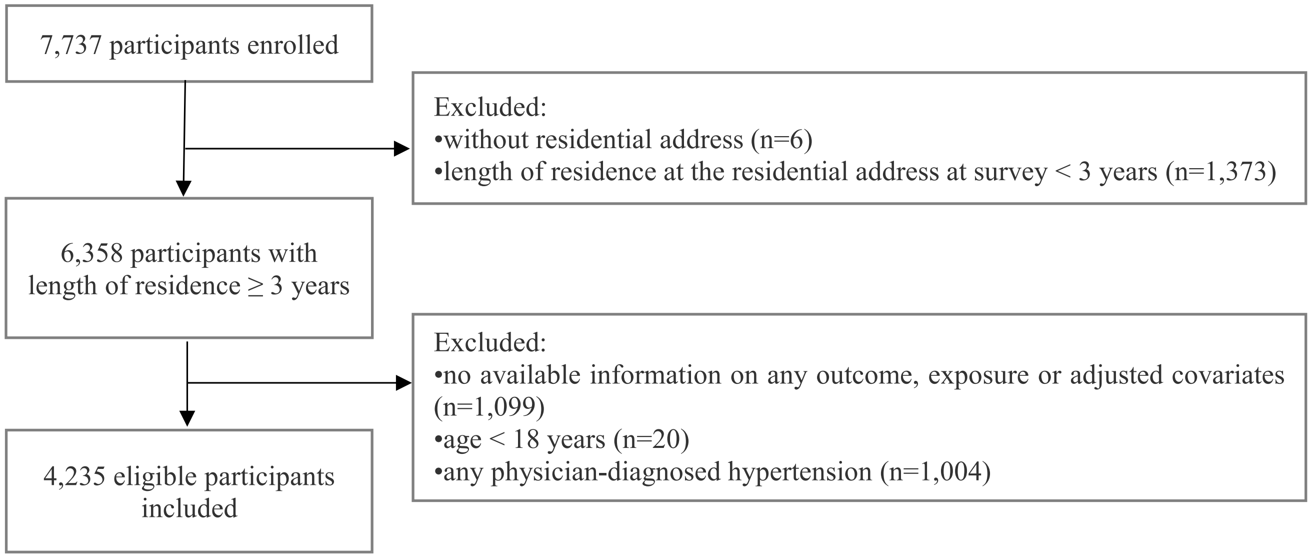

Figure S1 Flowchart of the study population.

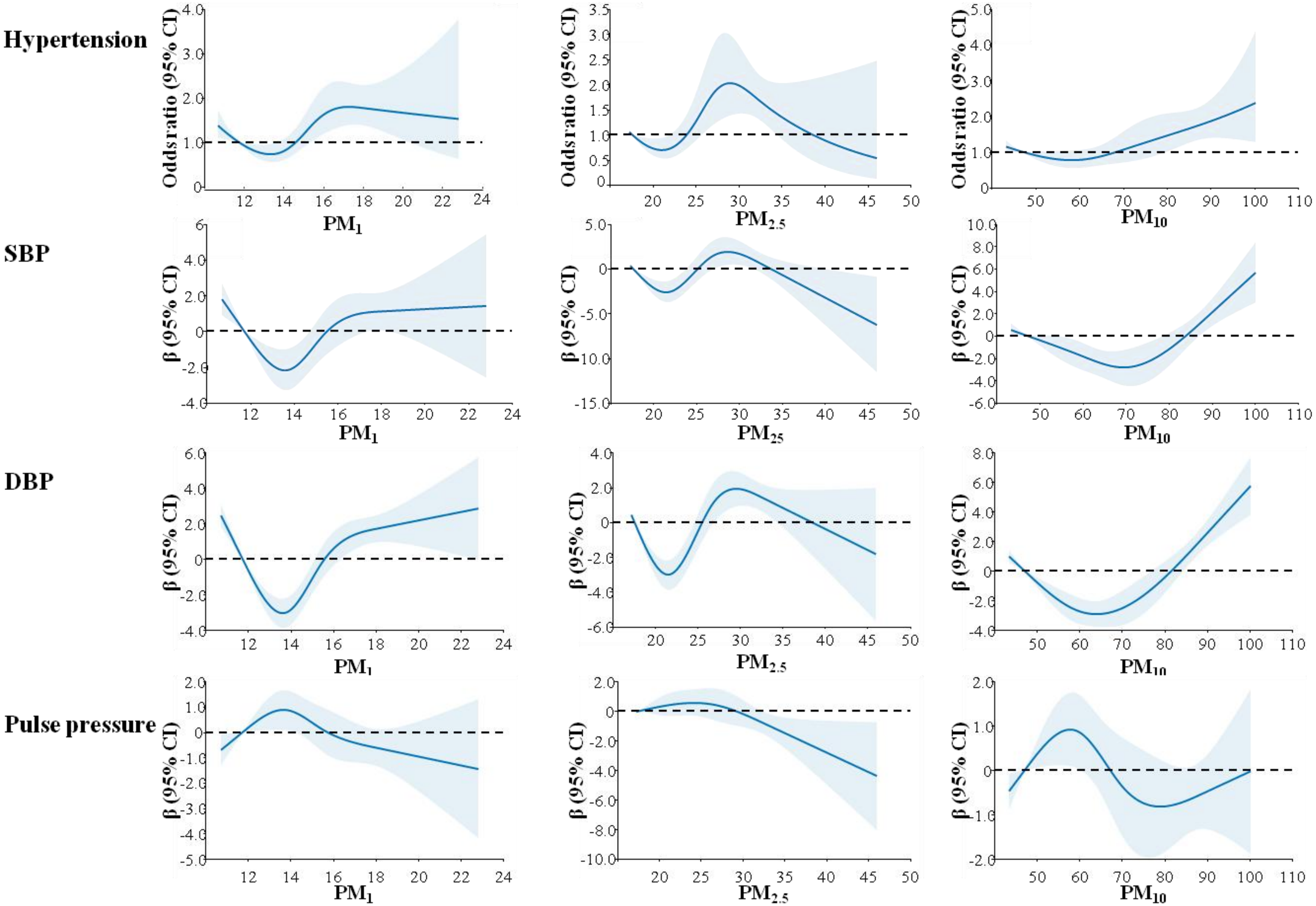

Hypertension
SBP
DBP
Pulse pressure
Oddsratio (95% CI)
β (95% CI)
PM1
PM2.5
PM10

Figure S2 Odds ratio, β and its 95%CI (confidence interval) for hypertension, SBP, DBP, and pulse pressure with ambient air pollutant exposure from the restricted splines regression model.

Models were adjusted for age, sex, marital, annual family income, educational level, smoking, secondary smoking, alcohol drinking, physical activity, hypertension family history, BMI, DASH score and indoor air pollution. BMI indicates body mass index; DASH, dietary approaches to stop hypertension; PM_1, the particle with an aerodynamic diameter of 1 μm or less; $PM_{2.5}$, the particle with an aerodynamic diameter of 2.5 μm or less; PM_{10}, the particle with an aerodynamic diameter of 10 μm or less; SBP, systolic blood pressure; DBP, diastolic blood pressure.

Table S1. Associations of risk of hypertension with per 10 μg/m^3 increase in ambient air pollutants in the sensitivity analyses

Outcome	Exposure	Model A	Model B
		OR (95CI)	OR (95CI)
Hypertension	PM_1	2.897(1.917, 4.377)	2.827(1.841, 4.341)
	$PM_{2.5}$	1.554(1.309, 1.844)	1.548(1.296, 1.849)
	PM_{10}	1.191(1.119, 1.267)	1.196(1.118, 1.279)
		β (95CI)	β (95CI)
DBP	PM_1	4.016(2.792, 5.241)	3.976(2.660, 5.292)
	$PM_{2.5}$	1.705(1.194, 2.216)	1.841(1.295, 2.388)
	PM_{10}	0.684(0.498, 0.870)	0.738(0.534, 0.942)
SBP	PM_1	2.500(0.799, 4.201)	2.357(0.526, 4.188)
	$PM_{2.5}$	0.843(0.133, 1.553)	0.978(0.216, 1.739)
	PM_{10}	0.451(0.192, 0.709)	0.482(0.197, 0.766)
PP	PM_1	-1.504(-2.666, -0.342)	-1.601(-2.858, -0.344)
	$PM_{2.5}$	-0.856(-1.340, -0.371)	-0.856(-1.379, -0.334)
	PM_{10}	-0.231(-0.408, -0.055)	-0.254(-0.449, -0.058)

Model A was adjusted for age, sex, marital, annual family income, educational level, smoking, secondary smoking, alcohol drinking, physical activity, hypertension family history, BMI, DASH score, indoor air pollution, temperature and humidity. Model B was adjusted for age, sex, marital, annual family income, educational level, smoking, secondary smoking, alcohol drinking, physical activity, hypertension family history, BMI, DASH score, indoor air pollution, and diabetes.

BMI, body mass index; DASH, dietary approaches to stop hypertension; PM_1, the particle with an aerodynamic diameter of 1 μm or less; $PM_{2.5}$, the particle with an aerodynamic diameter of 2.5 μm or less; PM_{10}, the particle with an aerodynamic diameter of 10 μm or less; SBP, systolic blood pressure; DBP, diastolic blood pressure; PP: pulse pressure; OR: odds ratio; CI: confidence interval.

Table S2. ORs and 95% CI for hypertension risk associated with per 10 μg/m^3 increase in ambient air pollutants

Outcome	Exposure	One-year	Two-year	Four-year
		OR (95CI)	OR (95CI)	OR (95CI)
Hypertension	PM_1	2.746(1.956, 3.854)	2.892(2.033, 4.114)	3.096(2.085, 4.596)
	$PM_{2.5}$	1.573(1.353, 1.830)	1.569(1.350, 1.824)	1.595(1.359, 1.873)
	PM_{10}	1.203(1.136, 1.274)	1.202(1.136, 1.273)	1.205(1.137, 1.277)
		β (95CI)	β (95CI)	β (95CI)
DBP	PM_1	4.278(3.231, 5.325)	4.460(3.370, 5.550)	4.187(2.980, 5.393)
	$PM_{2.5}$	1.959(1.495, 2.423)	1.940(1.478, 2.403)	1.884(1.393, 2.374)
	PM_{10}	0.828(0.653, 1.003)	0.814(0.640, 0.989)	0.790(0.614, 0.966)
SBP	PM_1	2.796(1.348, 4.243)	2.865(1.359, 4.372)	2.758(1.093, 4.422)
	$PM_{2.5}$	1.157(0.515, 1.798)	1.114(0.474, 1.753)	1.066(0.387, 1.744)
	PM_{10}	0.568(0.326, 0.810)	0.547(0.305, 0.789)	0.542(0.298, 0.786)
PP	PM_1	-1.470(-2.455, -0.484)	-1.581(-2.607, -0.556)	-1.415(-2.548,-0.282)
	$PM_{2.5}$	-0.797(-1.233, -0.360)	-0.821(-1.256, -0.387)	-0.812(-1.274, -0.351)
	PM_{10}	-0.258(-0.423, -0.093)	-0.266(-0.430,-0.101)	-0.246(-0.412,-0.080)

OR (95%CI) were adjusted for age, sex, marital, annual family income, educational level, smoking, secondary smoking, alcohol drinking, physical activity, hypertension family history, BMI, DASH score and indoor air pollution. BMI, body mass index; DASH, dietary approaches to stop hypertension; PM_1, the particle with an aerodynamic diameter of 1 μm or less; $PM_{2.5}$, the particle with an aerodynamic diameter of 2.5 μm or less; PM_{10}, the particle with an aerodynamic diameter of 10 μm or less; SBP, systolic blood pressure; DBP, diastolic blood pressure; PP: Pulse pressure; OR: odds ratio; CI: confidence interval.
